# Trends and Age-Period-Cohort Effect on Incidence of Male Breast Cancer from 1980 to 2019 in Taiwan and the USA

**DOI:** 10.3390/cancers16020444

**Published:** 2024-01-19

**Authors:** Jhao-Yang Peng, Yu-Kwang Lee, Rong-Qi Pham, Xiao-Han Shen, I-Hui Chen, Yong-Chen Chen, Hung-Shu Fan

**Affiliations:** 1 Graduate Institute of Business Administration, Fu Jen Catholic University, No. 510, Zhongzheng Rd., Xinzhuang Dist., New Taipei City 242062, Taiwan; 409088178@mail.fju.edu.tw; 2 Roche Diagnostics Ltd., Taipei City 10491, Taiwan; 3 Division of General Surgery, Department of Surgery, National Taiwan University Hospital, No. 7, Chung Shan S. Rd. (Zhongshan S. Rd.), Zhongzheng Dist., Taipei City 100225, Taiwan; viviankwang125@ntuh.gov.tw; 4 Institute of Public Health, National Yang Ming Chiao Tung University, No. 155, Sec. 2, Linong St., Beitou Dist., Taipei City 112304, Taiwan; yumi0427.md12@nycu.edu.tw; 5 Master Program of Big Data in Biomedicine, College of Medicine, Fu Jen Catholic University, No. 510, Zhongzheng Rd., Xinzhuang Dist., New Taipei City 242062, Taiwan; 411496092@m365.fju.edu.tw; 6 MacKay Memorial Hospital, No. 92, Sec. 2, Zhongshan N. Rd., Zhongshan Dist., Taipei City 104217, Taiwan; emily@mmh.org.tw; 7 School of Medicine, Fu Jen Catholic University, No. 510, Zhongzheng Rd., Xinzhuang Dist., New Taipei City 242062, Taiwan; 8 Data Science Center, College of Medicine, Fu Jen Catholic University, No. 510, Zhongzheng Rd., Xinzhuang Dist., New Taipei City 242062, Taiwan

**Keywords:** male breast cancer, age-period-cohort model, incidence

## Abstract

**Simple Summary:**

Although male breast cancer (MBC) is globally rare, its incidence significantly increased from 1990 to 2017. The aim of this study was to examine variations in the trends of MBC incidence between populations in Taiwan and the USA. Analysis of related databases from the two countries showed MBC incidence in Taiwan rapidly increased in contrast to the much slower increase in the USA during the same period. Furthermore, the results of the age-period-cohort analysis demonstrated a stronger cohort effect on the MBC incidence trend in Taiwan than in the USA. The observed cohort effect in this study is similar to that of a prior investigation on female breast cancer in Taiwan, indicating the potential influence of common environmental factors in both genders, such as a high-fat diet and xenoestrogen.

**Abstract:**

Although male breast cancer (MBC) is globally rare, its incidence significantly increased from 1990 to 2017. The aim of this study was to examine variations in the trends of MBC incidence between populations in Taiwan and the USA from 1980 to 2019. The Taiwan Cancer Registry database and the Surveillance, Epidemiology, and End Results (SEER) Program of the National Cancer Institute of the USA were used. The age-standardized incidence rate was calculated using the world standard population in 2000. The long-term trends of the age, time period, and birth cohort effect on MBC incidence rates were estimated using the SEER Age-Period-Cohort Web Tool. The results revealed that the incidence of MBC in both countries increased from 2010 to 2019 (Taiwan: average annual percentage change (AAPC) = 2.59%; USA: AAPC = 0.64%). The age and period effects on the incidence rates in both countries strengthened, but the cohort effect was only identified in Taiwan (Rate ratio: 4.03). The identified cohort effect in this study bears resemblance to that noted in a previous investigation on female breast cancer in Taiwan. This suggests the possible presence of common environmental factors influencing breast cancer incidence in both genders, such as a high fat diet and xenoestrogen.

## 1. Introduction

Female breast cancer is the most commonly diagnosed cancer (11.7% of total new patients) and is ranked globally in the top five causes of mortality of both sexes combined (6.9% of total mortality cases) [1]. Because the incidence of male breast cancer (MBC) is substantially lower than that of female breast cancer, MBC has been mostly ignored [2,3,4]. The age-standardized incidence rate of MBC globally increased from 0.46 per 10^5^ individuals in 1990 to 0.61 per 10^5^ individuals in 2017, and the average annual percentage change was 1.17% (95% confidential interval (95% CI): 1.01, 1.34). Notably, in East Asia, the age-standardized incidence rate has exceeded the global average, ranking first in terms of percentage increase [5]. Related MBC research must be conducted to study this difference in East Asia, including in Taiwan.

The risk factors for MBC include constitutional (aging) [6], environmental (radiation) [7], hormonal (abnormalities in estrogen/androgen balance) [7,8,9,10], obesity [7], and genetic (positive family history, Klinefelter syndrome, and mutations in BRCA1 and specifically in BRCA2) [11,12,13] factors. Moreover, when compared to female breast cancer (FBC), the age distribution among men diagnosed with breast cancer exhibits a solitary peak at 71 years. In contrast, for women, the distribution is marked by dual peaks at 52 and 71 years [14]. The Pearson correlation coefficient (r) for age-adjusted male and female breast cancer incidence rates across different countries from 1988 to 2002 in all ages was 0.69 (95% CI: 0.41, 0.85), suggesting that similarities may exist in the risk factors for breast cancer between sexes [6]. The results of the age-period-cohort analyses revealed a significant cohort effect on female breast cancer incidence in Taiwan [15], suggesting a higher relative risk (RR = 7.29) than in the USA (RR = 1.37). Although the factors contributing to differences in female breast cancer epidemiology have been extensively studied [16,17], MBC epidemiology in Taiwan remains understudied.

Since the 1960s, Taiwan has undergone significant industrialization, leading to a notable shift in lifestyle, such as Westernized ways of life. This may bring obesity and other factors which are known risks of MBC; so, the aim of this study was to examine the differences in long-term trends, from 1980 to 2019, of MBC incidence in populations in Taiwan and the USA.

## 2. Materials and Methods

We conducted an observational study of male patients diagnosed with breast cancer from 1980 to 2019 using the database of the Taiwan Cancer Registry (TCR) (http://www.doh.gov.tw/, accessed on 1 July 2023). The Ministry of Health and Welfare of Taiwan (former name was Department of Health of Taiwan) established the TCR in 1979. In Taiwan, the population-based cancer registry collects information on newly diagnosed cancer patients in hospitals with 50 or more beds throughout the country. According to the relevant law, every cancer case must be registered [18]. The TCR data completeness rate increased from 91.28% to 98.10%, from 2001 to 2020 [19,20]. The registry was reformed in 2002 to include details such as stage at diagnosis and the first course of treatment (called long-form database) to monitor cancer care patterns, evaluate cancer treatment outcomes, and to provide an essential foundation for academic research and cancer control policy in Taiwan [19]. In the TCR, each histological type is coded using the International Classification of Diseases for Oncology Field Trial Edition (ICD-O-FT) and the International Classification of Diseases for Oncology (ICD-O-3). Since 2002, the ICD-O-3 codes have been used. The ICD-O-FT morphology code for MBC is 174, and the ICD-O-3 site code for MBC is C50.0-C50.9. MBC epidemiological data and pathology results during the same period in the USA were collected from the Surveillance, Epidemiology, and End Results (SEER) Program of the U.S. National Cancer Institute (http://seer.cancer.gov/, accessed on 1 July 2023). We obtained permission to access the SEER database and extracted the data of 4157 MBC patients from the Incidence-SEER Research Data, 8 Registries, November 2022 Sub (1975–2020) using SEER*Stat software, version 8.4.1. The inclusion criteria were as follows: (1) the primary site of a malignant tumor was restricted to “breast (ICD-O-3 site code for MBC was C50)”; (2) sex was “male”; (3) the criterion for age is between 30 years old to 79 years old; (4) and complete clinical, surgical, pathological, and follow-up data were available.

## 3. Statistical Analysis

The age-specific incidence rates in Taiwan and the USA were calculated by calendar period and birth cohort. Age and calendar period were divided into 10-year groups, which resulted in five age intervals (30–39, 40–49, 50–59, 60–69, 70–79) and four period intervals (1980–1989, 1990–1999, 2000–2009, 2010–2019), respectively. Birth cohort was defined by determining the midpoint between the age and period interval, resulting in eight birth cohort interval (1905–1914, 1915–1924, 1925–1934, …, 1965–1974, 1975–1984) groups. To enable a direct comparison of rates across different countries, we utilized the World Health Organization (WHO) 2000 World Standard Population to calculate the age-standardized incidence rates (ASR) [21].

The Age-Period-Cohort Web Tool, developed by the SEER program of the USA National Cancer Institute (available at https://analysistools.cancer.gov/apc/, accessed on 1 August 2023) [22], was utilized for age-period-cohort modeling to assess period and cohort effects in both Taiwan and the USA. Additionally, the tool was employed to analyze the annual percentage change (APC) in expected age-specific rates (local drifts) and age-adjusted rates (Net drift), referred to as the average annual percentage change (AAPC), over a 20-year period. The tool provided point estimates along with 95% confidence intervals for these evaluations.

The input data included cases of age groups between 30 and 79 years, periods between 1980 and 2019, and cohorts between 1910 and 1989. The period and cohort effects are presented as incidence rate ratios. The hormone receptor is classified into three groups: both estrogen receptor-negative (ER−) and progesterone receptor-negative (PR−), referred to as ‘both negative’; both estrogen receptor-positive (ER+) and progesterone receptor-positive (PR+), termed ‘both positive’; and exclusively either estrogen receptor-positive (ER+) or progesterone receptor-positive (PR+), known as ‘neither positive’. 

## 4. Results

**Figure 1:**
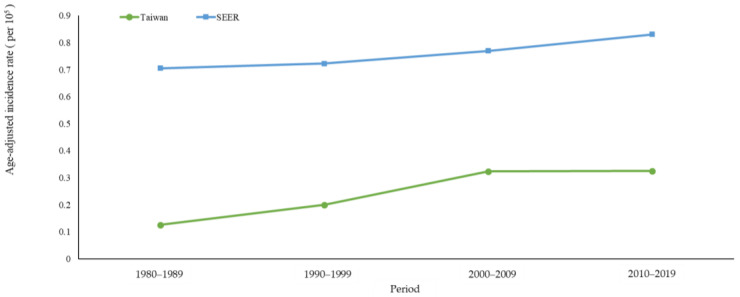
Age-standardized incidence rates of male breast cancer in Taiwan and the USA between 1980 and 2019.

Figure 1 shows the age-standardized incidence rates of MBC in Taiwan and the USA, and Table 1 displays the AAPC and APC for both countries across various age groups. The age-standardized incidence rate in Taiwan increased from 0.13 per 10^5^ individuals in 1980–1989 to 0.33 in 2010–2019 (AAPC = 2.59%, 95% CI: 1.64, 3.54), and from 0.71 to 0.83 per 10^5^ individuals in the USA during the same period (AAPC = 0.64%, 95% CI: 0.20, 1.08) (Table 1). The increase in the age-standardized incidence rate of MBC was greater in Taiwan than in the USA.

**Figure A1:**
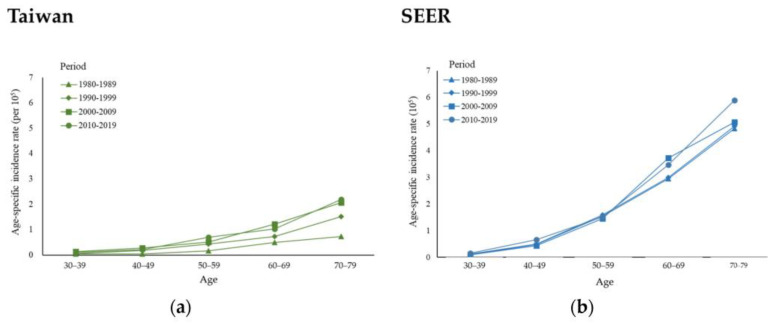
Age-specific incidence rates of male breast cancer in (**a**) Taiwan and (**b**) the USA between 1980 and 2019 by calendar period and age group.

Appendix A Figure A1 illustrates the age-specific incidence of MBC in Taiwan and the USA from 1980 to 2019, which indicates that in different periods, the incidence increased with age in both Taiwan and the USA. Taking 2010 to 2019 as an example, the incidence in Taiwan per 10^5^ individuals was 0.10 in the 30–39-year-old age group and reached 2.20 in the 70–79-year-old age group; incidences in the USA for these age groups were 0.14 and 5.89 per 10^5^ individuals, respectively.

A comparison of the APCs of different age groups shows that in Taiwan, MBC incidence increased more in older people, while the incidence in the USA was more stable. The APC was 2.89%/year (95% CI: 1.25, 4.57) in Taiwan and 0.69%/year (95% CI: 0.15, 1.24) in the USA for those aged 70 to 79 (Table 1).

**Figure A2:**
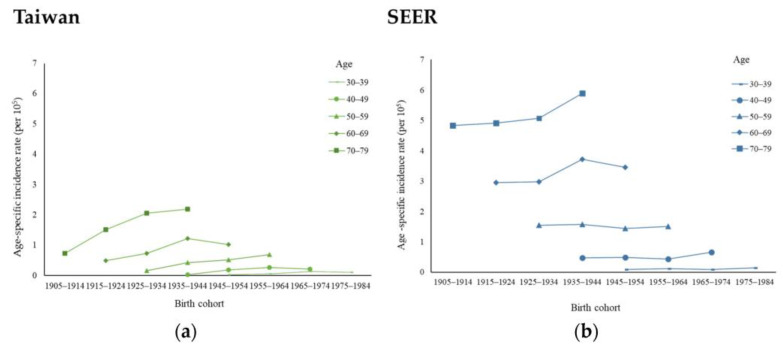
Cohort-specific incidence rates of male breast cancer in (**a**) Taiwan and (**b**) the USA between 1980 and 2019 by age group.

Appendix A Figure A2 depicts the cohort-specific incidence rate of MBC from 1980 to 2019. The incidence rates in both countries increased in the early cohorts and decreased in the later cohorts, with a slight difference noted between Taiwan and the USA. Compared with the USA, the upward trend in the cohort-specific MBC incidence in Taiwan was more pronounced. Taking the group aged 70 to 79 years as an example, the MBC incidence rate in Taiwan per 10^5^ individuals rose from 0.74 in 1905 to 1914 birth cohort, to 2.20 in the 1935 to 1944 birth cohort. In the USA, per 10^5^ individuals, MBC occurred at a rate of 4.83 in the 1905 to 1914 birth cohort, rising to 5.89 in the 1935 to 1944 birth cohort.

**Figure 2:**
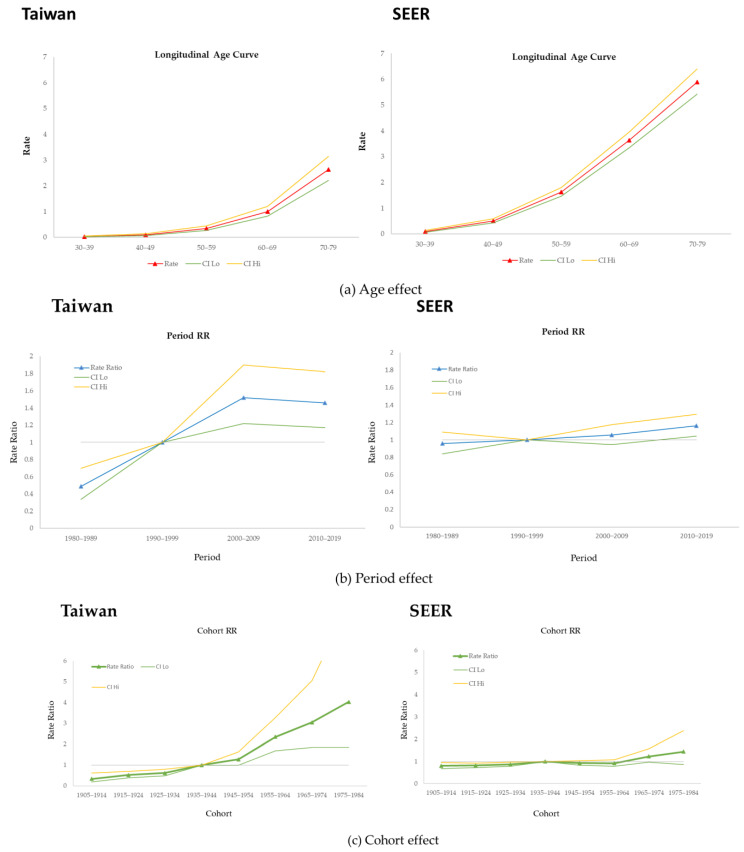
Age-period-cohort effect of male breast cancer in Taiwan and the USA.

Figure 2 shows the results of the APC analysis of MBC in Taiwan and the USA from 1980 to 2019. As depicted in Figure 2a, the age effect increased with age in Taiwan, with a longitudinal rate of 0.03 (95% CI: 0.02, 0.05) for those aged 30 to 39 years, to 2.63 for those aged 70 to 79 years (95% CI: 2.21,3.15). In the USA, the age effect strongly increased from 0.10 (95% CI: 0.07, 0.13) in the group aged 30–34, to 5.89 (95% CI: 5.42, 6.41) in those aged 70–79.

Estimation of the period effect revealed slight changes in both countries. The relative risk of MBC in Taiwan was 1.46 (95% CI: 1.17, 1.82) from 2010 to 2019; in the USA, it was 1.16 (95% CI: 1.04, 1.30) during the same period (Figure 2b).

Figure 2c shows the cohort effect in both countries. We used the 1940 birth cohort as the reference group to estimate this effect on populations in Taiwan and the USA. In Taiwan, the cohort effect exhibited a strong upward trend, reaching a maximum of 4.03 (95% CI: 1.85, 8.80) in 1980. For the USA, the curve was stable, and the value neared 1.0000. The highest value was 1.44 (95% CI: 0.87, 2.39) in 1980, and all cohorts showed nonsignificant differences.

**Figure 3:**
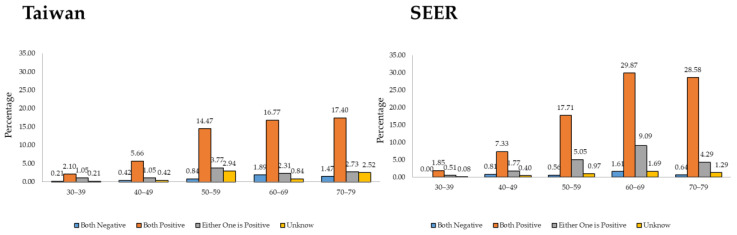
ER/PR status in Taiwan and the USA from 2010 to 2019.

Figure 3 shows both countries’ estrogen receptor (ER) and progesterone receptor (PR) rates for MBC status. In Taiwan, the ER- and PR-negative rates were less than 2% in all age groups, and the ER- and PR-positive rates trended upward with age. Similarly, in the USA, the ER- and PR-negative rates were less than 2% in all age groups, indicating that the MBC hormone receptor levels were similar between the countries.

## 5. Discussion

In this study, the MBC incidence in Taiwan rapidly increased throughout the studied 39 year period in contrast to the much slower increase in the USA during the same period. We also observed that the MBC incidence rates for Taiwan and the USA in different periods increased with age. However, MBC incidence rates among Taiwanese men born after the 1960s increased more compared with the rates among United States men. Furthermore, the results of the APC analysis demonstrated a stronger cohort effect on the MBC incidence trend in Taiwan than in the USA.

This study revealed that the MBC incidence rates in the USA were higher than in Taiwan. Compared with the rates in Japan and Korea, incidence rates in the USA are higher (Japan: 0.18% for 1988–2002; Korea: 0.2% for 1993–2002) [23,24]. The APC of MBC in Taiwan was higher than that in the USA. Compared with Asian countries, Taiwan also has a higher APC of MBC than Japan (APC: 1.06%, 95% CI: −2.24, 4.47) from 1988 to 2002 [6] and Korea (APC: −0.69%) from 1999 to 2016 [25]. Despite similarities in the age at diagnosis of patients in Taiwan, Japan, and Korea [25,26], the higher APC in Taiwan suggests the involvement of additional environmental factors in the increased MBC risk.

In an earlier study conducted in the USA, a case-control approach was employed to identify the risk factors of MBC; the findings indicated that obesity, limited exercise, and consuming red meat were associated with elevated MBC risk [7]. Prior research has also shown obesity to be a known risk factor for MBC [7,27,28,29,30]. Taiwan has become increasingly industrialized since the 1960s, and the main difference experienced by the younger compared with the older generations in Taiwan is the increasingly Westernized lifestyle. A previous study using the Nutrition and Health Survey in Taiwan of 1993 to 1996 showed the prevalence rates of overweightness and obesity were 22.9 and 10.5% for males [30]. Men born after the 1960s have been exposed to more high-calorie and high-fat diets in their childhood and generally have a higher body mass index than previous cohorts. Therefore, obesity could be a possible factor contributing to the notable cohort effect observed in the MBC incidence trend in Taiwan.

Some pre-clinical studies have presented evidence indicating a positive link between breast cancer development and the intake of a high-fat diet. These investigations suggest that a high-fat diet leads to the deposition of adipose tissue in the mammary glands and that obesity triggers a pro-inflammatory response, creating a conducive microenvironment for breast cancer carcinogenesis [31,32,33]. Studies involving humans have revealed a connection between a high-fat diet and the development of breast cancer, although the findings from human trials were not as pronounced as those observed in preclinical studies [34,35]. However, in a 2014 research endeavor encompassing approximately 300,000 participants across Europe, aimed at investigating various connections between lifestyle factors and cancer, including dietary patterns, findings were published that established an association between HER2-negative breast cancer and hormone receptor-positive breast cancer, with a particular emphasis on saturated fats [36].

Prior research suggests that the exposure to common chemicals plays a role in the rising incidence of breast cancer [37]. Industrialization has increased the number of manufactured products used in everyday life, such as polybrominated diphenyl ethers, bisphenol A, phthalates, and alkylphenol polyethoxylates (APEOs). These products cause people to be environmentally exposed to hormones, and environmental hormones are a risk factor in the occurrence of cancer [37,38,39,40,41]. APEOs, for example, comprise a class of nonionic surfactants commonly used as emulsifiers, detergents, pesticides, and wetting and foaming agents in various industrial, agricultural, and household applications [42]. A total of 650,000 tons of APEOs are produced globally every year [43]. Approximately 60% of APEOs are discharged annually into aquatic environments [44,45], which degrade into shorter-chain and more stable alkylphenols, such as nonylphenol (NP) and octylphenol (OP) [46]. However, more than 130,000 metric tons of APEOs were used in 1997, in Taiwan [47]. A previous study showed that in Taiwan, APEOs were detected in 41% of 90 household detergents at concentrations ranging from 0.2% to 21% [48]. Because less than 10% of all municipal wastewater was treated in wastewater treatment plants in Taiwan at that time, the alkylphenol concentrations would have been higher in Taiwanese rivers and sediments than in other countries [49]. Given that people in Taiwan might have been exposed to high concentrations of alkylphenols, alkylphenols might increase the risk of MBC [50,51]. Additionally, in 1988, the Construction and Planning Agency of the Ministry of the Interior devised the Sewerage Development Plan for wastewater. Given the widespread use of large amounts of APEOs in Taiwan, individuals born in the 1970s and 1980s may experience exposure to APEOs/bisphenol at older ages within this specific birth-year cohort compared to previous birth-year cohorts.

The positive rates of ER and PR in Taiwan were lower than those in the USA, but they exhibited an increasing trend with age. Studies have noted a positive association exclusively between ER- and PR-positive breast cancer and Western dietary patterns [52,53]. Additionally, experimental animal data and epidemiological studies have extensively documented the endocrine-disrupting effects of certain environmental estrogen-like chemicals in humans. Using bisphenol A as an example, exposure produces changes in mammary gland development, modifications in gene expression within the mammary gland, and an increased expression of estrogen receptor-α (ERα) and PRs [54,55]. The ER/PR status of MBC cases in Taiwan may indicate that environmental hormones and Western dietary patterns contribute to the cohort effects of MBC in the Taiwanese population.

Although numerous researchers have used the age-period-cohort model to analyze the incidence trend of female breast cancer [14,56,57], studies employing the age-period-cohort model to examine the trend in MBC incidence and explore potential environmental factors influencing MBC incidence are scarce. This study showed a strong cohort effect similar to that reported in a previous Taiwanese study [15], which indicated similar environmental factors for breast cancer incidence in both sexes in the country.

Numerous genes have been recognized as contributing to a risk of more than double in developing familial breast cancer. These genes include BRCA1, BRCA2, CHEK2, PALB2, BRIP1, PTEN, ATM, and TP53 [58]. Mutations in BRCA1 and BRCA2 make up around 15% of female breast cancer (FBC). In MBC, BRCA2 mutations are more prevalent than BRCA1 mutations and carry a lifetime relative risk for developing the disease of approximately 8.9% [59]. In the previous studies, the frequency of BRCA2 mutations in MBC varied from 4 to 40% [60,61,62,63]. In the United States, Dr. Yuan’s research revealed that out of 115 MBC cases, 18 exhibited mutations in BRCA2, representing a ratio of 15.6% [64]. In Taiwan, there has been no prior investigation addressing BRCA2 mutations in male breast cancer (MBC). Nonetheless, based on a previous study, the findings indicated a 8.6% mutation rate in BRCA2 exon 11 [65].

This study has several strengths. First, we used a population-based and highly representative database to analyze the long-term trend in MBC incidence in Taiwan. Second, the study covered a period of almost 39 years, and we used the age-period-cohort model to evaluate period and cohort effects to identify possible exogenous factors influencing MBC incidence. This study has some limitations. First, due to the considerably lower incidence of MBC, we employed 10-year intervals as the framework for computing incidence rates. The use of longer 10-year intervals may potentially overlook or dismiss certain data points. Second, the TCR database does not include personal-level data of patients with cancer, so we lacked some information including family history, social history, BRCA1/BRCA2 mutations, comorbidities, and personal history of cancer, which are factors known to affect MBC incidence. The age-period-cohort model has often been used to analyze time trends in carcinoma incidence, but trend studies based on histological types are lacking. In the present study, we analyzed the long-term trend in MBC incidence according to age, period, and birth cohort in Taiwan and the USA. The results provide information about the underlying causal mechanisms of MBC and provide a reference for future research.

## 6. Conclusions

Our results showed some similarities in the incidence of MBC between Taiwan and the USA, with the incidence increasing with age in both countries. However, we also revealed noticeable differences between the countries, with Taiwan having a strong cohort effect not found in the USA. The observed cohort effect in this study is similar to that of a prior investigation on female breast cancer in Taiwan, indicating the potential influence of common environmental factors in both genders, such as a high-fat diet and xenoestrogen.

## Figures and Tables

**Table 1:** Net drift and local drift of male breast cancer by age group in Taiwan and the USA between 1980 and 2019.

		Taiwan			SEER		
		%	95% CI		%	95% CI	
AAPC		2.59	1.64	3.54	0.64	0.20	1.08
APC	30–39 years	1.86	–0.56	4.34	1.61	0.06	3.19
	40–49 years	3.30	1.49	4.60	0.62	–0.16	1.39
	50–59 years	3.16	1.93	4.41	0.10	–0.43	0.63
	60–69 years	2.03	0.98	3.09	0.51	0.09	0.94
	70–79 years	2.89	1.25	4.57	0.69	0.15	1.24

AAPC, average annual percentage change; APC, annual percentage change; CI, confidence interval.

## Data Availability

MBC cases between 1980 and 2019 were obtained from Taiwan’s Cancer Registry System and the Surveillance, Epidemiology, and End Results (SEER) Program of the National Cancer Institute of the USA.

## Abbreviations

MBC	Male Breast Cancer
SEER	Surveillance, Epidemiology, and End Results
AAPC	Average Annual Percentage Change
TCR	Taiwan Cancer Registry
ICD-O-FT	International Classification of Diseases for Oncology Field Trial Edition
ICD-O-3	International Classification of Diseases for Oncology
WHO	World Health Organization
ASR	Age-Standardized Incidence Rates
APC	Annual Percentage Change
ER−/+	Estrogen Receptor-Negative/Positive
PR−/+	Progesterone Receptor-Negative/Positive
APEOs	Alkylphenol Polyethoxylates
NP	Nonylphenol
OP	Octylphenol
FBC	Female Breast Cancer
